# Evaluation of the phenological, pomological, physicochemical, and biochemical characteristics of early peach and nectarine cultivars grown under mediterranean conditions

**DOI:** 10.1186/s12870-026-09731-z

**Published:** 2026-08-11

**Authors:** Sabriye Atmaca

**Affiliations:** Department of Plant and Animal Production, Department of Organic Agriculture, Akdeniz University, Manavgat Vocational School, Antalya, 07600 Türkiye

**Keywords:** *Prunus persica* L. Batsch, *Prunus persica* L. Batsch var nectarina, Early cultivar, Phenological stages, Sugar, Phenols, Chemical composition

## Abstract

**Background:**

Peach is an important summer fruit widely cultivated in temperate and subtropical regions of the world. This research was conducted between 2021 and 2025 at the experimental and research orchard of Akdeniz University, Manavgat Vocational School (Manavgat, Antalya, Türkiye). In the study, the flowering phenology as well as the pomological, physicochemical, and biochemical characteristics of early peach (Filomena, Plogod-9) and nectarine (Garbaja, Patagonia) cultivars were evaluated.

**Results:**

It was determined that the phenological stages of early peach and nectarine cultivars generally exhibited similarities depending on climatic conditions. According to the average values, bud break occurred between late January and early February (21 January–6 February), flowering took place between late January and mid-February (27 January–20 February), and full bloom was observed between early February and early March (6 February–5 March). Harvesting was completed during May (7 May–30 May). In all cultivars, leaf fall began around 15 November and continued until 15 December.

In early peach cultivars, the average fruit weight was determined to range from 66.50 to 121.20 g, fruit width from 46.56 to 57.78 mm, fruit length from 50.12 to 62.35 mm, seed weight from 4.86 to 8.35 g, seed width from 16.45 to 20.13 mm, and seed length from 27.24 to 34.98 mm. In early nectarine cultivars, the average fruit weight ranged from 77.60 to 119.90 g, fruit width from 49.98 to 60.85 mm, fruit length from 44.04 to 57.24 mm, seed weight from 5.35 to 8.69 g, seed width from 16.51 to 20.01 mm, and seed length from 24.84 to 31.87 mm.

Among the peach cultivars, Filomena exhibited higher values, particularly in terms of soluble solids content (SSC) and certain color parameters (a* and C*), whereas Plogod-9 showed higher L and h° values in some years. These findings indicate that fruit quality attributes may vary depending on the year. Among the nectarine cultivars, Garbaja was found to have higher values for most color parameters (L, a*, b*, C*, and h°).

As a result of biochemical analyses, the Patagonia cultivar was found to stand out in terms of phenolic compounds and total sugar content, while the Garbaja cultivar exhibited higher flavonoid content, and the Plogod-9 cultivar showed higher values in terms of vitamin C content.

**Conclusion:**

In conclusion, it was demonstrated that early peach and nectarine cultivars with low chilling requirements can be successfully grown under Mediterranean conditions. Furthermore, the studied early cultivars were found to possess significant potential in terms of biochemical characteristics, and it was concluded that these cultivars are promising for early-season production in the Mediterranean ecology.

## Introduction

Peaches (*Prunus persica* L. Batsch) and nectarines (*Prunus persica* L. Batsch var. nectarina [Ait] Maxim.) rank as the third most economically important fruit crops worldwide after apple (*Malus* spp.) and pear (*Pyrus* spp.) [1–3]. According to FAO 2023 data, global peach and nectarine production covers approximately 1,561,641 ha, with a total production of 27,077,873.01 tons [4]. Countries located in temperate climate zones, particularly China, Italy, the United States, Spain, Greece, and Türkiye, are among the leading producers of peaches and nectarines worldwide [3, 5]. In Türkiye, FAO 2025 data indicate that peach and nectarine production is carried out on 55,928 ha, with a total production of 1,076,852 tons [4]. Peach and nectarine fruits are primarily consumed fresh; however, they are also used in processed products such as fruit juice, jam, and yogurt, and to a lesser extent, as dried fruit [3].

Fruit development duration generally ranges between two and nine months and covers the period from full bloom to harvest. Peach genotypes are classified according to fruit development duration as early (≤ 90 days), mid-season (91–125 days), or late-ripening (> 125 days) groups. Although genetic factors play a determining role in peach fruit maturation, it is also significantly influenced by environmental conditions [6]. Flowering phenology is considered a fundamental biological trait for plant adaptation to environmental changes and for maintaining reproductive success. Phenological processes, through biological activities, enable the assessment of the impacts of climate change on individual plant fitness and biodiversity [7]. Detailed examination of phenological developmental stages in peach trees can serve as an important tool for planning disease and pest management strategies at different growth stages [8].

In addition to being highly preferred by consumers due to its sensory attributes, peach is also regarded as a rich source of bioactive compounds [9]. Peach fruit contains various bioactive compounds, including phenolic compounds and vitamin C, as well as carbohydrates, organic acids, carotenoids, and flavonoids, all of which contribute to increasing its nutritional value and consumer acceptability [3, 10–15]. The major sugar fractions in peach include sucrose, fructose, sorbitol, and glucose. The proportions of these sugars vary throughout the fruit ripening process; glucose and fructose are relatively higher during the early stages of ripening, whereas sucrose content increases markedly as ripening progresses. At full maturity, sucrose becomes the dominant sugar component in peach fruit [1, 2, 12, 16]. In different studies, it has been reported that the sugar, organic acid, vitamin, and phenolic compound contents of peach and nectarine fruits show considerable variation depending on the geographical regions in which they are grown, climatic conditions, and genotypic differences [3, 9–12, 14, 17–22].

Studies conducted in different ecological regions have shown that local and standard cultivars exhibit significant differences in terms of yield, pomological, and biochemical characteristics, highlighting the necessity of region-specific cultivar selection [5, 20, 23–35]. The Mediterranean Region of Türkiye provides suitable areas for early peach and nectarine cultivars with low chilling requirements [32]. Evaluating the performance of newly developed low-chilling cultivars under different growing conditions is of great importance for achieving both earliness and high fruit quality objectives.

This study aimed to evaluate the flowering phenology as well as the pomological, physicochemical, and biochemical characteristics of early peach (Filomena, Plogod-9) and nectarine (Garbaja, Patagonia) cultivars with low chilling requirements grown under the ecological conditions of the Mediterranean Region of Türkiye, and to determine their performance, thereby providing a scientific reference based on the obtained findings.

## Materials and methods

### Experimental material and site

This study was conducted between 2021 and 2025 in Antalya (Manavgat), Türkiye, at the Ilıca experimental and research field of the Organic Agriculture Program of Akdeniz University Manavgat Vocational School (36°48’31.08” N and 31°23’16.47” E, 24 m above sea level). The soil texture of the experimental site was sandy loam (47% sand, 21% clay, and 32% silt), with a soil pH of 7.8 and a lime content of 40.3%. The analyses were conducted at the Regional Soil, Plant, Water and Fertilization Analysis Laboratory of the Western Mediterranean Agricultural Research Institute.

Climatic data were obtained from the nearest meteorological station to the experimental site, station number MANAVGAT/17,954, belonging to the General Directorate of Meteorology under the Ministry of Environment, Urbanization and Climate Change. Daily maximum, minimum, and mean temperature and humidity values were recorded. Monthly minimum temperatures ranged from 0.30 to 22.8 °C, mean temperatures from 9.62 to 31.63 °C, and maximum temperatures from 20.50 to 44.7 °C (Table 1). Relative humidity values were determined to range from 25.0 to 76.0% for minimum values, 43.7–74.3% for mean values, and 87.0–99.0% for maximum values (Table 2).

**Table 1:** Monthly maximum, minimum, and mean temperature values recorded at the experimental site during the study period

Year/ Month	Maximum Temperature											
	1	2	3	4	5	6	7	8	9	10	11	12
2023	21.8	25.6	25.9	28.6	32.1	38.9	44.7	39.0	32.6	30.5	30.5	24.7
2024	22.6	23.2	29.7	33.5	32.6	44.7	43.4	42.3	32.4	30.6	30.6	21.5
2025	20.6	20.7	31.0	28.9	34.3	39.3	-	-	-	-	-	-
	Mean Temperature											
2023	12.9	10.9	15.2	16.8	20.9	25.5	31.6	29.5	27.4	23.3	18.6	15.0
2024	13.6	13.9	15.7	20.8	22.1	29.7	31.5	30.4	27.1	22.7	17.1	13.2
2025	13.3	10.2	16.2	17.1	21.6	27.3	-	-	-	-	-	-
	Minimum Temperature											
2023	4.9	3.6	6.2	9.3	11.3	16.8	21.9	21.4	18.8	15.4	5.6	8.8
2024	5.7	7.4	7.6	11.1	12.5	18.1	22.7	22.8	18.5	11.0	5.5	7.2
2025	5.9	0.8	6.7	6.0	11.8	13.3	-	-	-	-	-	-

**Table 2:** Monthly maximum, minimum, and mean relative humidity values recorded at the experimental site during the study period

Year/ Month	Maximum Relative Humidity											
	1	2	3	4	5	6	7	8	9	10	11	12
2023	99	96	99	97	99	93	94	93	99	95	99	99
2024	99	99	95	99	99	92	88	96	92	88	99	99
2025	99	99	96	99	97	99	-	-	-	-	-	-
	Mean Relative Humidity											
2023	62.9	48.2	63.9	64.2	66.1	62.8	45.9	65.7	52.9	55.9	63.6	67.1
2024	63.6	64.2	58.3	60.2	63.4	55.3	52.4	59.1	59.1	48.4	54.0	74.3
2025	66.9	46.7	56.5	65.9	69.3	56.1	-	-	-	-	-	-
	Minimum Relative Humidity											
2023	66	33	45	67	70	39	31	75	51	59	49	65
2024	33	55	45	57	63	42	42	64	63	26	39	54
2025	62	31	38	61	76	50	-	-	-	-	-	-

In this study, early peach cultivars Filomena and Plogod-9 and early nectarine cultivars Garbaja and Patagonia (PSB Producción Vegetal, Spain), obtained from a commercial company and characterized by low chilling requirements, were used. The cultivars were grafted onto Garnem (GN15) rootstock, and the trees were established on 04.05.2021 with a planting spacing of 5.0 × 3.0 m. A goblet training system was applied to the trees. A drip irrigation system was used for water supply. Adjustable emitters commonly used in orchards (0–100 L h⁻¹) were installed at the base of each plant. Irrigation was initiated in the summer period following the end of rainfall and continued until September–October, when rainfall resumed. During the summer months, irrigation was applied once or twice a week depending on air temperature, whereas in spring it was applied approximately every 10 days according to plant water requirements. Throughout the growing period, regular weed control and pest and disease management practices were carried out.

### Evaluation of phenological parameters

In this study, bud swelling (bud break) (% bud burst), defined as the date when the highest level of bud break per plant was observed, was recorded; flowering onset (when approximately 5% of flowers were open), full bloom (when approximately 80% of flowers were open), end of flowering (when more than 90% of flowers were open), harvest time (when fruits attained firmness, color, and taste characteristics), the number of days from full bloom to harvest, fruit set percentage (%), and leaf fall period (when leaves were completely shed) were determined according to Demiral [36].

### Evaluation of pomological parameters

For the determination of pomological parameters, measurements were performed on eight fruits randomly selected from each tree for each cultivar. Pomological parameters included fruit weight (g), fruit width (mm), fruit length (mm), seed weight (g), seed width (mm), seed length (mm), fruit skin color values (L, a*, b*, C*, h°), and total soluble solids content (%). Fruit and seed weights were measured in grams using a precision balance. The width and length of the fruits, as well as the width and length of the seeds, were measured in millimeters using a digital caliper. Fruit skin color was determined using a 3NH-NR60CP colorimeter by measuring L, a*, b*, C* and h° values at three equally spaced points around the equatorial region of each fruit. L represents lightness, a* indicates redness/greenness, b* indicates yellowness/blueness, C* represents chroma (color saturation), and h° represents hue angle. Total soluble solids content (%) was determined using a digital refractometer (HANNA HI96801). Measurements were performed on juice extracted from fruits using a fruit juicer.

### Biochemical analyses

Within the scope of biochemical analyses, total phenolic content (mg GAE/100 g sample), total flavonoid content (mg KE/100 g sample), sugar composition (g/100 g; sucrose, glucose, and fructose), and vitamin C content (mg/100 g) were determined using randomly selected fruits harvested in 2025 from each cultivar. All analyses were carried out at the Akdeniz University Food Safety and Agricultural Research Center.

#### Analysis of total phenols in extracts

For the extraction of phenolics, one gram of sample of pulps from each group was accurately weighed into a flask, and 20 mL of aqueous ethanol (60%, containing 0.1% HCL) was added. The phenolics were extracted in a water bath at 120 rpm and 30 °C for 30 min. The supernatant was collected and stored at 4 °C until the analysis was performed [37].

The concentration of total phenols in extracts was carried out according to the Folin-Ciocalteu method (Škerget et al. 2005). For this, a 0.5 mL diluted extract was mixed with 2.5 mL Folin-Ciocalteu reagent (0.1 N) and 2 mL Na_2_CO_3_ (7.5%, w/V), respectively. Then the mixture was incubated at 50 °C for 5 min and immediately cooled. The absorbance of the samples was recorded at 760 nm wavelength with a spectrophotometer (Shimadzu, UV-Vis 160 A, Japan) against a blank solution (prepared with 60% aqueous ethanol). The results were calculated via a calibration curve (0-100 ppm GA) and expressed as gallic acid equivalent (mg GAE/ 100 g).

#### Analysis of total flavonoid content

The determination of the total flavonoid content was carried out according to the method used by Dinçer [38]. For this purpose, 0.5 mL diluted extract was mixed with 2.5 mL distilled water, 150 µL NaNO_2_ (5%, w/V), 300 µL AlCl_3_ (10%, w/V), and 1 mL NaOH (1 N), respectively. After adding each solution, the mixture was vortexed and allowed to stand for 5 min. The final volume was made up to 5 mL with distilled water. The absorbance of the samples was measured at 510 nm using a spectrophotometer (Shimadzu UV-Vis 160 A, Japan) against a blank solution prepared with aqueous methanol (80%). The results were calculated via a calibration curve (0-100 ppm) and expressed as catechin equivalent (mg CE/ 100 g).

#### Ascorbic acid analysis

The determination of total ascorbic acid content was carried out according to the method used by Asami et al. [39]. For the ascorbic acid analysis, firstly samples were homogenized with waring blender. 1–5 g sample were transferred to centrifuge tubes and ascorbic acid were extracted with 20 mL metaphosphoric acid (4.5%). Then the mixture was centrifuged at 1650 *g* for 15 min at 20 °C, supernatant filtered through 0.45 μm membrane filter and injected to (20 µL) HPLC system (CBM-20 A controller, LC 20AD pump, SIL-20 A autosampler, CTO-20 A column oven, SPD-M20A PDA detector, Shimadzu, Japan). The elution carried put with a 5 μm reversed-phase C18 column (4.6 × 250 mm) with a flow rate of 0.5 mL/min using ultra-pure water (pH 2.2, adjusted with sulphuric acid) as mobile phase. The column maintained at 25 °C and detection wavelength was 245 nm. The amount of ascorbic acid was calculated with an external calibration curve, and method validation was performed using the standard addition method.

#### Sugar analysis (sucrose, glucose, fructose)

The sugar composition analysis was carried out according to the Tetik et al. [40]. To this end, 2 g of samples were extracted with ultra-pure water (1:25, w/V). The diluted samples were filtered with a 0.45 μm membrane filter prior to the injection (20 µL) to the HPLC system (CBM-20 A controller, LC 20AD pump, SIL-20 A autosampler, CTO-20 A column oven, RID-10 A detector, Shimadzu, Japan). The elution of sugars was conducted with a reversed-phase HPLC column (CARBOsep Coregel 87P, 7.8 × 300 mm^2^) at 85 °C. Ultra-pure water used as mobile phase at a flow rate of 0.6 mL/min. the concentrations of sucrose, glucose and fructose were calculated using external standard calibration curves prepared from standard solutions (25–250 mg/L). Method validation was performed using the standard addition method.

### Data evaluation and statistical analyses

The experiment was conducted using a completely randomized design (CRD). Experimental plots, each consisting of 12 plants of a single cultivar, were randomly arranged within the experimental area. Data were collected from all 12 plants in each plot, and each plant was considered an independent experimental unit and biological replicate (*n* = 12). No subsampling was performed. Since the study included two peach cultivars and two nectarine cultivars, statistical comparisons were performed separately within each species (peach and nectarine). Thus, only two cultivars were compared in each analysis, and differences between cultivar means were determined using Student’s t-test (*P* < 0.05). Because considerable interannual climatic variation was observed during the study period, data from each year were analyzed independently. Statistical analyses were performed using Minitab 17.1.0.0 statistical software.

For the biochemical analyses (phenolic compounds, flavonoids, sugars, and vitamin C), a single composite sample was prepared for each cultivar and analyzed once (*n* = 1). Therefore, measures of variability (standard deviation or standard error) could not be calculated, and no statistical comparisons among cultivars were performed.

## Results and discussion

### Evaluation of phenological parameters

In phenological parameters, marked differences were observed among years depending on climatic conditions, whereas no significant differences were found among cultivars over the years (Table 3). Based on the phenological observations in 2023, bud break started on 6 February, full bloom occurred between 24 and 28 February, and flowering ended on 7 March.

**Table 3:** Dates of bud break, initiation of bloom, full bloom, end of bloom, and leaf fall recorded in early peach cultivars Filomena and Plogod-9 and early nectarine cultivars Garbaja and Patagonia

Variety	Year	Bud break	Initiation of bloom	Full bloom	End of bloom	Leaf fall
Filomena	2023	6 February	15 February	24–28 February	7 March	15 November-15 December
	2024	31 January	20 February	28 February − 5 March	12 March	15 November-15 December
	2025	21 January	27 January	6 February	19 February	15 November-15 December
Plogod-9	2023	6 February	15 February	26 February	7 March	15 November-15 December
	2024	31 January	20 February	28 February − 5 March	12 March	15 November-15 December
	2025	21 January	27 January	6 February	19 February	15 November-15 December
Garbaja	2023	6 February	15 February	26 February	7 March	15 November-15 December
	2024	31 January	20 February	28 February − 5 March	12 March	15 November-15 December
	2025	21 January	27 January	6 February	19 February	15 November-15 December
Patagonia	2023	6 February	15 February	26 February	7 March	15 November-15 December
	2024	31 January	20 February	28 February − 5 March	12 March	15 November-15 December
	2025	21 January	27 January	6 February	19 February	15 November-15 December

In 2024, higher minimum temperatures influenced phenological development, and bud break occurred on 31 January, approximately one week earlier than the previous year. Full bloom was observed between 28 February and 5 March, and flowering ended on 12 March. Accordingly, bud break occurred earlier in 2024; however, the full bloom period was distributed over a wider time range.

In 2025, bud break occurred on 21 January, representing the earliest date among the studied years. Full bloom was recorded on 6 February, and the end of flowering was observed on 19 February. These findings indicate a notably early and rapid phenological progression in 2025.

Autumn temperatures did not show significant variation among the years, and leaf fall occurred between 15 November and 15 December in all cultivars.

When the harvest dates were evaluated across years, it was observed that significant differences emerged depending on climatic conditions (Table 4). In 2023, harvest occurred on May 17 for the peach cultivars and on May 30 for the nectarine cultivars, which may be associated with the relatively stable temperature conditions during the spring period. In 2024, higher average temperatures recorded in March and particularly in April accelerated vegetative development. As a result, harvest dates advanced by approximately 10–16 days in all cultivars, and harvest was carried out on May 7 for the peach cultivars and on May 14 for the nectarine cultivars. In contrast, in 2025, despite the relatively high temperatures observed in March, the decrease in temperatures during April together with the decline of minimum temperatures to critical levels slowed phenological development and delayed harvest dates. Consequently, harvest took place on May 9 for the peach cultivars and on May 20 for the nectarine cultivars in 2025.

**Table 4:** Harvest date, days from full bloom to harvest, and fruit set rates determined for the early peach cultivars Filomena and Plogod-9, and the early nectarine cultivars Garbaja and Patagonia

Variety	Year	Harvest date	Days from full bloom to harvest	Fruit set ratios (%)
Filomena	2023	17.05.2023	80	53.93
	2024	7.05.2024	68	56.29
	2025	9.05.2025	90	11.01
Plogod-9	2023	17.05.2023	80	39.15
	2024	7.05.2024	68	54.33
	2025	9.05.2025	90	19.65
Garbaja	2023	30.05.2023	93	42.27
	2024	14.05.2024	75	49.67
	2025	20.05.2025	103	15.24
Patagonia	2023	30.05.2023	93	42.43
	2024	14.05.2024	75	29.71
	2025	20.05.2025	103	1.96

When days from full bloom to harvest (DFBH) was evaluated across years, significant differences were observed (Table 4). In 2023, this period was determined as 80 days in the peach cultivars and 93 days in the nectarine cultivars. In 2024, however, phenological development accelerated due to the high temperatures observed during March and April. Accordingly, days from full bloom to harvest shortened to 68 days in the peach cultivars and 75 days in the nectarine cultivars. In contrast, in 2025, the development process slowed particularly due to temperature fluctuations and low minimum temperatures during the post-bloom period, resulting in a longer period from full bloom to harvest, reaching 90 days in the peach cultivars and 103 days in the nectarine cultivars.

There is a clear relationship between fruit set and climatic conditions (Table 4). In 2023 and 2024, the moderate temperatures and intermediate humidity conditions provided a favorable environment for flowering and fertilization processes, thereby contributing to high fruit set rates. Although the mean March temperature in 2025 was relatively high (16.24 °C), the decrease in temperature in April (17.16 °C) and, in particular, the decline of minimum temperatures to critical levels (February: 0.8 °C; April: 6.0 °C) created significant cold stress and frost risk during the flowering period. These adverse conditions directly affected pollination and fruit set processes, leading to a significant reduction in fruit set rates. Indeed, a marked decline in fruit set was observed across all cultivars in 2025; for instance, in the ‘Patagonia’ cultivar, fruit set decreased from 42% to 29% to 1.96%, while in the ‘Filomena’ cultivar it declined from 56% to 11%. Based on these findings, 2025 was evaluated as a problematic year in which stress conditions predominated during the flowering period.

When the responses of the cultivars to climatic conditions were examined, it was observed that the peach cultivars Filomena and Plogod-9 were able to largely maintain their yields despite climatic stress, indicating a higher level of adaptability. In contrast, the nectarine cultivars Garbaja and Patagonia were found to be more sensitive to environmental conditions. In particular, a continuous decline in yield was observed over the years in the Garbaja cultivar, whereas marked fluctuations were evident in the Patagonia cultivar. Indeed, the reduction of fruit set to as low as 1.96% in 2025 in the Patagonia cultivar clearly demonstrates its high sensitivity to adverse climatic conditions.

Based on the temperature data from 2023 to 2025 and the phenological observations of early peach (Filomena, Plogod-9) and nectarine (Garbaja, Patagonia) cultivars, it is clearly evident that interannual temperature variations were decisive in shaping flowering phenology. While phenological differences among cultivars remained relatively limited, the more pronounced year-to-year variations indicate that phenological development is largely driven by environmental conditions.

These findings are also consistent with the results of studies conducted under different ecological conditions. In a study carried out in the Mornag region of Tunisia, it was reported that full bloom and harvest dates in early, mid-, and late-season cultivars varied significantly across years, and that this variation was closely associated with chilling accumulation [25]. Similarly, it was reported that in northern subtropical conditions of India, peach cultivars with low chilling requirements flowered in February (8–20 February), and harvest was completed between late April and mid-May [27]. Huang et al. [5] also reported that, depending on cultivar and year, the 50% bloom date varied between 6 and 27 February, while the 50% leaf fall date ranged from 7 November to 13 December. Studies conducted under subtropical conditions indicate that interannual variation in winter chilling accumulation plays a decisive role in flowering onset and duration in low-chill cultivars; under insufficient chilling conditions, flowering periods are prolonged and floral synchrony is disrupted, whereas in years with adequate chilling accumulation, flowering occurs in a more compact and uniform manner [41]. Likewise, Huang et al. [5], in a study conducted under subtropical conditions in Taiwan with low-chill cultivars, reported that low winter chilling accelerated phenological development; however, cultivar-dependent responses were observed depending on adaptive capacity. In several studies on peach and nectarine, it has been reported that cultivars failing to meet their chilling requirements exhibit problems such as irregular flowering, reduced yield, and poor fruit quality [28, 42, 43].

Studies conducted under the ecological conditions of Hatay/Türkiye also reveal similar results. It has been reported that full bloom in early peach and nectarine cultivars occurred in the third week of February [28], while in different early nectarine cultivars, flowering was completed between late February (27 February) and early March (4 March), with harvest taking place in early June (7–11 June) [32].

It has been reported that the period from full bloom to harvest in early peach and nectarine cultivars generally ranges between 60 and 90 days, and in most early genotypes this period occurs in less than 90 days [44]. This enables early cultivars to reach the market earlier due to their short developmental periods. Srivastava et al. [27] reported that, under subtropical conditions, the period from full bloom to harvest in early peach cultivars ranged between 62 and 89 days. Çalışkan et al. [32] reported that, in nectarine cultivars, the number of days from full bloom to harvest showed significant variation among years; this period was 84–86 days in 2018, 104–109 days in 2019, 94–97 days in 2020, and 102 days across all cultivars in 2021.

Researchers emphasized that flowering and harvest times are not solely dependent on genetic characteristics; rather, interannual variations in chilling accumulation and temperature conditions during the growing period exert a decisive influence on phenological development [32].

### Evaluation of pomological parameters

#### Fruit weight, fruit width, and fruit length

The mean fruit weight, fruit width, and fruit length values determined for the early peach cultivars Filomena and Plogod-9 in 2023, 2024, and 2025 are presented in Table 5. Over the years examined, statistically significant differences were detected among the cultivars in terms of certain pomological characteristics. In particular, in 2024, the Plogod-9 cultivar exhibited higher values of fruit weight, fruit width, and fruit length compared to the Filomena cultivar. In contrast, in 2025, the Filomena cultivar showed higher values in terms of fruit weight and fruit width. In 2023, while differences were observed between cultivars in fruit width, fruit weight and fruit length values were found to be similar.

**Table 5:** Mean fruit weight, fruit width, and fruit length values determined in the early peach cultivars Filomena and Plogod-9 in 2023, 2024, and 2025

	Fruit weight (g)		Fruit width (mm)		Fruit length (mm)	
	Filomena	Plogod-9	Filomena	Plogod-9	Filomena	Plogod-9
2023	66.50 ± 1.80	70.60 ± 1.80	46.56 ± 0.52 b	48.62 ± 0.48 a	50.20 ± 0.49	50.27 ± 0.46
2024	105.90 ± 2.60 b	121.20 ± 2.80 a	54.44 ± 0.64 b	57.78 ± 0.61 a	58.27 ± 0.54 b	62.35 ± 1.20 a
2025	80.20 ± 2.30 a	70.50 ± 1.30 b	50.59 ± 0.60 a	48.70 ± 0.34 b	50.76 ± 0.80	50.12 ± 0.35

Each year was evaluated separately using a t-test

Different letters (a–b) were used to indicate statistically significant differences

The mean fruit weight, fruit width, and fruit length values determined for the early nectarine cultivars Garbaja and Patagonia in 2023, 2024, and 2025 are presented in Table 6. In the first year, the Patagonia cultivar exhibited higher values of fruit weight, fruit width, and fruit length compared to the Garbaja cultivar, and these differences were found to be statistically significant (*p* < 0.01). In the second year, no statistically significant differences were detected between the cultivars for all investigated pomological traits. In the third year, while no significant differences were found between the cultivars in terms of fruit weight and fruit width, a statistically significant difference (*p* < 0.01) was observed for fruit length.

**Table 6:** Mean fruit weight, fruit width, and fruit length values determined in the early nectarine cultivars Patagonia and Garbaja in 2023, 2024, and 2025

	Fruit weight (g)		Fruit width (mm)		Fruit length (mm)	
	Garbaja	Patagonia	Garbaja	Patagonia	Garbaja	Patagonia
2023	77.60 ± 2.40 b	89.30 ± 3.10 a	49.98 ± 0.70 b	60.85 ± 1.20 a	49.38 ± 0.57 b	53.87 ± 1.10 a
2024	112.80 ± 3.90	119.50 ± 5.10	57.09 ± 0.75	59.10 ± 0.94	57.24 ± 0.95	55.54 ± 0.96
2025	100.50 ± 3.90	92.70 ± 5.00	56.98 ± 0.86	55.81 ± 1.10	50.84 ± 0.80 a	44.04 ± 0.85 b

Each year was evaluated separately using a t-test

Different letters (a–b) were used to indicate statistically significant differences

Overall, the Patagonia cultivar stood out particularly in the first year in terms of fruit size; however, the differences between cultivars decreased over the years, and in some traits the Garbaja cultivar showed superior performance. This indicates that cultivar performance may vary depending on the year.

Fruit weight is considered one of the most important physical quality criteria determining yield, market value, and consumer preferences in peach and nectarine production. Studies have shown that fruit weight can vary considerably depending on genetic factors (cultivar), rootstock, ecological conditions, and cultural practices applied [14].

In the literature, considerable variation in fruit weight and fruit size has been reported in early peach and nectarine cultivars grown under different ecological conditions. Under Spanish conditions, lower fruit weights were observed in very early cultivars [45], whereas in subtropical conditions of India, fruit weight in early peach cultivars was reported to range between 34.85 and 80.57 g [27]. In studies conducted in Türkiye, it has been reported that fruit weight and fruit size values in cultivars such as Astoria, Maya, Garbaja, Gardeta, Gartario, and Garofa are generally consistent with the findings of the present study [28, 32]. However, in some cultivars, higher or lower values have also been reported depending on ecological conditions and genetic differences [26, 35]. These variations are considered to result from cultivar characteristics, growing conditions, and environmental factors.

In early peach cultivars, fruit weight is generally reported to be lower compared with mid- and late-season cultivars, consistent with a general biological trend [6, 8, 20, 46, 47]. This is attributed to the fact that late-season cultivars remain on the tree for a longer period and consequently accumulate more assimilates [35]. In addition, it has been reported that many pomological traits, particularly fruit weight, may show considerable year-to-year variability [24].

#### Seed weight, seed width, and seed length

The mean seed weight, seed width, and seed length values determined for the early peach cultivars Filomena and Plogod-9 in 2023, 2024, and 2025 are presented in Table 7. In all years examined, the Filomena cultivar exhibited higher values of seed weight and seed width compared to the Plogod-9 cultivar, and this difference was found to be statistically significant (*p* < 0.01). Regarding seed length, in the first year the Filomena cultivar showed higher and statistically significant values; in the second year, the differences between cultivars were found to be statistically insignificant. In the third year, however, a significant difference was observed between cultivars in terms of seed length, with higher values recorded in the Plogod-9 cultivar.

**Table 7:** Mean seed weight, seed width, and seed length values determined in the early peach cultivars Filomena and Plogod-9 in 2023, 2024, and 2025

	Seed weight (g)		Seed width (mm)		Seed length (mm)	
	Filomena	Plogod-9	Filomena	Plogod-9	Filomena	Plogod-9
2023	5.55 ± 0.11 a	5.04 ± 0.09 b	17.32 ± 0.13 a	16.75 ± 0.16 b	29.55 ± 0.28 a	28.19 ± 0.30 b
2024	8.35 ± 0.15 a	7.24 ± 0.17 b	20.13 ± 0.15 a	19.18 ± 0.23 b	31.98 ± 0.39	31.28 ± 0.35
2025	5.91 ± 0.01 a	4.86 ± 1.03 b	18.05 ± 0.29 a	16.45 ± 0.14 b	27.24 ± 0.26 b	28.12 ± 0.49 a

Each year was evaluated separately using a t-test

Different letters (a–b) were used to indicate statistically significant differences

The mean seed weight, seed width, and seed length values determined for the early nectarine cultivars Garbaja and Patagonia in 2023, 2024, and 2025 are presented in Table 8. For seed weight, the Garbaja cultivar showed higher and statistically significant values (*p* < 0.05) compared with the Patagonia cultivar in all years. Regarding seed width, no significant difference was observed in 2023, whereas statistically significant differences (*p* < 0.01) were found in the second and third years. In terms of seed length, no significant differences were detected between the cultivars across years, and the values remained close to each other.

**Table 8:** Mean seed weight, seed width, and seed length values determined in the early nectarine cultivars Garbaja and Patagonia in 2023, 2024, and 2025

	Seed weight (g)		Seed width (mm)		Seed length (mm)	
	Garbaja	Patagonia	Garbaja	Patagonia	Garbaja	Patagonia
2023	6.57 ± 0.15 a	6.04 ± 0.20 b	18.15 ± 0.18	19.97 ± 0.90	29.25 ± 0.29	29.14 ± 0.62
2024	8.69 ± 0.25 a	7.52 ± 0.28 b	20.01 ± 0.22 a	18.42 ± 0.38 b	31.71 ± 0.56	31.87 ± 0.56
2025	6.66 ± 0.21 a	5.35 ± 0.26 b	19.20 ± 0.43 a	16.51 ± 0.39 b	26.11 ± 0.48	24.84 ± 0.84

Each year was evaluated separately using a t-test

Different letters (a–b) were used to indicate statistically significant differences

Seed weight and seed dimensions are among the pomological traits that directly influence the flesh-to-seed ratio in peach and nectarine cultivars and therefore play an important role in determining fruit market value. In the literature, wide variation in seed characteristics has been reported among different peach and nectarine cultivars. Under the conditions of Bursa/Türkiye, seed weight in standard peach cultivars was reported to range between 4.20 and 11.72 g [20], whereas in cultivars such as Astoria, Maya, and Garbaja, values between 7.42 and 10.08 g were reported [28]. Similarly, it has been reported that seed weight and seed dimensions in nectarine cultivars grown under Mediterranean climatic conditions are generally consistent with the findings of the present study [48, 49]. In the literature, it has been stated that seed characteristics in early peach and nectarine cultivars can vary significantly depending on genetic factors; in particular, seed weight and seed width show marked differences among cultivars, whereas variation in seed length is relatively limited [35, 50, 51]. Likewise, Çelik et al. [52] emphasized that seed weight and seed width in early nectarine cultivars are associated with fruit size and yield characteristics.

#### Color parameters (L, a*, b*, C*, h°)

The mean color (L, a*, b*, C*, h°) values determined for the early peach cultivars Filomena and Plogod-9 in 2023, 2024, and 2025 are presented in Table 9. Regarding the L value, in the second year the Plogod-9 cultivar showed a higher and statistically significant (*p* < 0.01) value, whereas no significant differences were detected between cultivars in the other years. For the a* value, the Filomena cultivar exhibited higher and statistically significant differences in the first and third years. No significant differences were observed between the cultivars in terms of b* values across the years. Regarding the C* (chroma) value, a statistically significant difference in favor of the Filomena cultivar was detected in the first year, whereas the cultivars showed similar values in the other years. For the h° (hue angle), the Plogod-9 cultivar exhibited higher and statistically significant values in the second and third years.

**Table 9:** Mean color (L, a*, b*, C*, h°) values determined in the early peach cultivars Filomena and Plogod-9 in 2023, 2024, and 2025

	L values		a* values		b* values		c* values		h* values	
	Filomena	Plogod-9	Filomena	Plogod-9	Filomena	Plogod-9	Filomena	Plogod-9	Filomena	Plogod-9
2023	38.74 ± 0.50	39.86 ± 0.57	26.61 ± 0.43a	25.25 ± 0.36b	18.26 ± 0.49	17.54 ± 0.45	32.70 ± 0.56a	31.15 ± 0.44b	33.08 ± 0.57	33.65 ± 0.66
2024	42.16 ± 0.69b	45.41 ± 0.57a	27.02 ± 0.38	26.13 ± 0.38	22.07 ± 0.64	23.17 ± 0.56	35.47 ± 0.52	35.50 ± 0.46	37.92 ± 0.85b	40.48 ± 0.78a
2025	37.26 ± 0.74	38.94 ± 0.43	26.15 ± 0.43a	24.39 ± 0.29b	16.77 ± 0.80	17.99 ± 0.42	31.44 ± 0.73	30.77 ± 0.41	31.07 ± 1.0b	34.92 ± 0.53a

Each year was evaluated separately using a t-test

Different letters (a–b) were used to indicate statistically significant differences

The mean color (L, a*, b*, C*, h°) values determined for the early nectarine cultivars Garbaja and Patagonia in 2023, 2024, and 2025 are presented in Table 10. Regarding the L value, the Garbaja cultivar exhibited higher and statistically significant (*p* < 0.01) values in all years. For a* and b* values, the Garbaja cultivar showed higher and statistically significant (*p* < 0.01) values in the first and third years. The C* (chroma) values were higher in the Garbaja cultivar in all years, and these differences were found to be statistically significant (*p* < 0.01). Regarding the h° (hue angle), the Garbaja cultivar exhibited higher and statistically significant (*p* < 0.01) values in the second and third years.

**Table 10:** Mean color (L, a*, b*, C*, h°) values determined in the early nectarine cultivars Garbaja and Patagonia in 2023, 2024, and 2025

	L values		a* values		b* values		c* values		h* values	
	Patagonia	Garbaja	Patagonia	Garbaja	Patagonia	Garbaja	Patagonia	Garbaja	Patagonia	Garbaja
2023	34.6 ± 0.73a	28.83 ± 0.63b	29.16 ± 0.35a	19.16 ± 0.65b	15.43 ± 0.67a	8.98 ± 0.59b	33.57 ± 0.48a	21.49 ± 0.77b	26.62 ± 0.99	24.03 ± 1.1
2024	44.41 ± 1.4a	37.24 ± 0.92b	29.05 ± 0.78	29.40 ± 0.64	24.35 ± 1.1a	18.53 ± 1.10b	39.47 ± 0.59a	35.39 ± 1.0b	38.1 ± 1.70a	30.37 ± 1.4b
2025	34.90 ± 0.60a	31.75 ± 0.36b	27.64 ± 0.50a	25.20 ± 0.66b	14.88 ± 0.73a	10.18 ± 0.56b	31.94 ± 0.71a	27.40 ± 0.80b	26.30 ± 0.94a	20.69 ± 0.70b

Each year was evaluated separately using a t-test

Different letters (a–b) were used to indicate statistically significant differences

Fruit color is one of the most important quality criteria in peaches and nectarines, directly influencing consumer preference and marketability. CIELAB color parameters such as skin lightness (L), redness (a*), yellowness (b*), color saturation (C*), and hue angle (h°) are shaped by the interaction of several factors, including genetic background, environmental conditions, cultivation system, fruit maturity stage, and harvest time.

The findings obtained in this study regarding fruit skin color were generally consistent with those reported in studies conducted under different ecological conditions and cultivars. It has been reported that in the Flariba nectarine cultivar grown under protected cultivation conditions, the highest L value was obtained under Mediterranean/Mersin conditions, whereas more intense red coloration developed under Samandağ/Hatay conditions [26]. Similarly, wide variation in L, h°, and C* values has been reported in nectarine cultivars grown in the Lapseki region [49]. It has been stated that fruit color characteristics are influenced by several factors, including cultivar, ecological conditions, cultural practices, fruit position within the canopy, and rootstock used [53]. In addition, it has been reported that interannual climatic differences, phenological development, and heat accumulation also have significant effects on fruit skin color [28, 32, 47, 54].

#### Soluble solid content (SSC, %)

The mean soluble solids content values determined for the early peach cultivars Filomena and Plogod-9 in 2023, 2024, and 2025 are presented in Table 11. Regarding soluble solids content (SSC), no statistically significant differences were observed between the cultivars in the first two years, whereas in the third year, the Filomena cultivar showed higher and statistically significant (*p* < 0.01) values compared with the Plogod-9 cultivar. In the Garbaja and Patagonia cultivars, the Patagonia cultivar exhibited higher and statistically significant (*p* < 0.01) values than the Garbaja cultivar in the first and third years.

**Table 11:** Soluble solids content (%) determined in the early peach cultivars Filomena and Plogod-9, and the early nectarine cultivars Garbaja and Patagonia

2023	Filomena	Plogod-9	Garbaja	Patagonia
	6.77 ± 0.14	6.80 ± 0.15	7.56 ± 0.22 b	8.99 ± 0.30 a
2024	9.59 ± 0.21	8.96 ± 0.25	10.65 ± 0.32	11.52 ± 0.44
2025	6.24 ± 0.24 a	5.10 ± 0.12 b	8.18 ± 0.31 b	9.99 ± 0.53 a

Each year was evaluated separately using a t-test.

Different letters (a–b) were used to indicate statistically significant differences.

Soluble solids content is an important component of peach fruit quality and is also considered a reliable indicator of fruit maturity [53]. It has been reported that SSC values in peach and nectarine cultivars vary depending on genetic background, ecological conditions, and cultivation practices [50, 52]. Previous studies have indicated that late-ripening cultivars generally tend to have higher SSC values compared with early-ripening cultivars [24].

The results obtained in this study were found to be consistent with those reported in studies conducted on early-ripening cultivars under similar ecological conditions [20, 28, 35, 48, 49, 55]. In contrast, lower SSC values were obtained compared with those reported in studies on late-ripening cultivars [32]. This may be attributed to the shorter fruit development period of early cultivars and their relatively lower sugar accumulation capacity.

In addition, based on the findings obtained, it was determined that SSC values were higher in all cultivars in 2024 compared with the other years. This is thought to be associated with the higher average temperatures recorded throughout 2024. Indeed, Sejfić et al. [48] reported that high temperature and low precipitation conditions promote an increase in SSC in fruits.

### Biochemical analyses

The total phenolic content, total flavonoid content, sugar composition, and vitamin C content determined in the early peach cultivars Filomena and Plogod-9 and the early nectarine cultivars Garbaja and Patagonia are presented in Table 12.

**Table 12:** Total phenolic content, total flavonoid content, sugar composition (g/100 g), and vitamin C content determined in the early peach cultivars Filomena and Plogod-9 and the early nectarine cultivars Garbaja and Patagonia

Variety	Total phenolic content (mg GAE/100 g )	Total flavonoid content (mg KE/100 g)	Sugar composition (g/ 100 g)			Vitamin C content (mg/100 g)
			Sakkaroz	Glukoz	Fruktoz	
Filomena	27.55	15.47	3.13	0.94	0.98	4.25
Plogod-9	19.51	12.31	1.97	0.66	0.76	6.11
Garbaja	22.41	16.52	2.32	0.72	0.81	3.48
Patagonia	41.76	14.92	3.43	0.79	0.92	5.93

Regarding total phenolic content, the Patagonia nectarine cultivar exhibited a total phenolic content of 41.76 mg GAE/100 g, followed by Filomena, Garbaja, and Plogod-9. Regarding total flavonoid content, Garbaja exhibited the numerically highest value (16.52 mg KE/100 g), whereas Plogod-9 had the numerically lowest value among the evaluated cultivars.

When the sugar composition was examined, it was observed that sucrose, glucose, and fructose contents showed a similar distribution across all cultivars; however, sucrose was generally the predominant sugar. In this context, the Patagonia nectarine cultivar exhibited the numerically highest sucrose content, whereas the Plogod-9 peach cultivar showed the numerically lowest sucrose content. Regarding vitamin C content, the Plogod-9 peach cultivar exhibited the numerically highest value (6.11 mg/100 g), while the Garbaja nectarine cultivar showed the numerically lowest value (3.48 mg/100 g).

In this study, total phenolic content, total flavonoid content, sugar composition, and vitamin C content were evaluated in the early peach cultivars Filomena and Plogod-9 and the early nectarine cultivars Garbaja and Patagonia. Regarding total phenolic content, the Patagonia nectarine cultivar exhibited the numerically highest value (41.76 mg GAE/100 g), which is consistent with previous studies reporting that phenolic compounds are largely genotype-dependent [19, 50, 51]. For total flavonoid content, the Garbaja cultivar exhibited the numerically highest value (16.52 mg KE/100 g). Mrázová et al. [12] similarly reported that phenolic and flavonoid contents vary widely among peach cultivars.

Regarding sugar composition, sucrose was identified as the predominant sugar in all cultivars. This finding is consistent with previous studies reporting sucrose as the main sugar component in peach and nectarine fruits [3, 9, 17, 35, 48, 53, 56]. However, Jayarajan et al. [19] reported fructose as the dominant sugar in some nectarine cultivars. These differences may be attributed to genetic background, ecological conditions, cultivation practices, and fruit maturity stage. In particular, it has been suggested that sucrose accumulation may be higher in early cultivars grown under warm Mediterranean conditions. In addition, it has been reported that environmental conditions and interannual climatic variability can significantly affect sugar accumulation [24, 28, 48].

Regarding vitamin C content, the ‘Plogod-9’ cultivar exhibited the numerically highest value (6.11 mg/100 g). It has been reported in the literature that vitamin C content in peach and nectarine cultivars may vary depending on genetic background and growing conditions [54, 57].

## Conclusions

In this study conducted under Mediterranean climatic conditions (Manavgat, Antalya, Türkiye), the phenological development stages, pomological characteristics, and physicochemical and biochemical properties of early peach and nectarine cultivars were evaluated. Within the scope of the study, critical phenological stages related to plant growth and development in peach and nectarine production under Mediterranean conditions were determined. The combined evaluation of phenological observations and quality parameters revealed that early cultivars performed successfully in the Mediterranean environment, and it was determined that high temperature conditions, in particular, promoted earliness, providing a significant advantage in terms of early marketability.

In the study, although interannual variations were observed in some quality attributes, particularly fruit size, the ability of the cultivars to largely maintain their yield and quality characteristics indicated a high level of adaptation to regional conditions. It was determined that the open-field peach cultivars Filomena and Plogod-9 and the nectarine cultivar Patagonia are promising genotypes for early-season cultivation under the regional conditions. In addition, the findings were considered to provide an important reference for future studies aimed at improving peach and nectarine production under protected cultivation systems.

Regarding fruit biochemical properties, the Patagonia cultivar exhibited the numerically highest total phenolic content and sucrose concentration, while the Garbaja cultivar exhibited the numerically highest flavonoid content and the Plogod-9 cultivar exhibited the numerically highest vitamin C content. In addition, sucrose was the predominant sugar based on the measured values in all cultivars. These results indicate that the evaluated early-ripening cultivars have potential not only in terms of early maturation traits but also with respect to nutritional value and functional properties.

When the responses of the cultivars to climatic conditions were evaluated, it was determined that the peach cultivars Filomena and Plogod-9 were able to largely maintain their yield despite climatic stress, indicating a higher level of adaptability. In contrast, the nectarine cultivars Garbaja and Patagonia were found to be more sensitive to environmental conditions. Overall, the results of this study demonstrated that the early peach and nectarine cultivars examined have considerable potential for early-season production under Mediterranean ecological conditions.

## Data Availability

The datasets generated and/or analysed during the current study are available from the corresponding author (Sabriye Atmaca; uysalsabriye@akdeniz.edu.tr) upon reasonable request.

## Abbreviations

CE: Catechin equivalents
CRD: Completely randomized design
DFBH: Days from full bloom to harvest
FAO: Food and Agriculture Organization of the United Nations
GAE: Gallic acid equivalents
HPLC: High-performance liquid chromatography
PDA: Photodiode array
SSC: Soluble solids content

## References

[1] Cantín CM, Gogorcena Y, Moreno MÁ. Analysis of phenotypic variation of sugar profile in different peach and nectarine [*Prunus persica* (L.) Batsch] breeding progenies. J Sci Food Agric. 2009;89(11):1909–17. 10.1021/jf900385a 19397288

[2] Font i Forcada C, Gradziel TM, Gogorcena Y, Moreno MA. Phenotypic diversity among local Spanish and foreign peach and nectarine [*Prunus persica* (L.) Batsch] accessions. Euphytica. 2014;197(2):261–77.

[3] Petruccelli R, Bonetti A, Ciaccheri L, Leri F, Ganino T, Faraloni C. Evaluation of the Fruit Quality and Phytochemical Compounds in Peach and Nectarine Cultivars. Plants. 2023;12:1618. 10.3390/plants12081618 PMC10144225 37111844

[4] FAO. Food and Agriculture Organization of the United Nations. FAOSTAT: Crops and livestock products. https://www.fao.org/faostat/en/#data/QCL. Accessed 31 December 2025.

[5] Huang CC, Wen IC, Lin SY, Tainung. 7 HongLing: A low-chill peach cultivar for early fresh market. HortScience. 2024;59(3):304–6.

[6] Maatallah S, Guizani M, Montevecchi G, Santunione G, Flamini G, Dabbou S. Investigation of pomological and biochemical properties of peach cultivars with different ripening periods. J Food Compos Anal. 2025;108427. 10.1016/j.jfca.2025.108427.

[7] Sun S, Frelich LE. Flowering phenology and height growth pattern are associated with maximum plant height, relative growth rate and stem tissue mass density in herbaceous grassland species. J Ecol. 2011;99(4):991–1000.

[8] Bhattarai N, Saud S, Atreya PN, Dhakal S, Bohara AK. Assessment of phenological and physicochemical characteristics of peach (*Prunus persica* L.) varieties in Mustang, Nepal. Archives Agric Environ Sci. 2024;9(4):793–9.

[9] Aubert C, Chalot G. Physicochemical characteristics, vitamin C, and polyphenolic composition of four European commercial blood-flesh peach cultivars (*Prunus persica* L. Batsch). J Food Compos Anal. 2020;86:103337.

[10] Nafti M, Khaldi Z, Haddad B. Phenotypic and biochemical diversity in peach [*Prunus persica* (L.) Batsch] cultivars. J New Sci. 2018;*51:2286–5314*.

[11] Shahkoomahally S, Chang Y, Brecht JK, Chaparro JX, Sarkhosh A. Influence of rootstocks on fruit physical and chemical properties of peach cv. UFSun Food Sci Nutr. 2021;9(1):401–13. 10.1002/fsn3.2005 PMC7802566 33473302

[12] Mrázová M, Rampáčková E, Šnurkovič P, Ondrášek I, Nečas T, Ercisli. Determination of selected beneficial substances in peach fruits. Sustainability. 2021;13(24):14028.

[13] Abidi W, Akrimi R. Phenotypic diversity of nutritional quality attributes and chilling injury symptoms in four early peach [*Prunus persica* (L.) Batsch] cultivars grown in west central Tunisia. J Food Sci Technol. 2022;59(10):3938–50. 10.1007/s13197-022-05425-0 PMC9525473 36193378

[14] Ciccoritti R, Manganiello R, Antonucci F, Ceccarelli D. Interactive Effect of Cultivars, Crop Years and Rootstocks on the Biochemical Traits of *Prunus persica* (L.) Batsch Fruits. Plants. 2023;12(12):2325. 10.3390/plants12122325 PMC10301400 37375950

[15] Usenik V, Ondrášek I, Petkovsek MM, Kastelec D. Compositional Data Analysis of the Chemical Composition in Prunus persica Fruit. J Food Compos Anal. 2025;107982. 10.1016/j.jfca.2025.107982.

[16] Guizani M, Maatallah S, Dabbou S, Serrano M, Hajlaoui H, Helal AN, Kilani-Jaziri S. Physiological behaviors and fruit quality changes in five peach cultivars during three ripening stages in a semi-arid climate. Acta Physiol Plant. 2019;41(9):154.

[17] Colarič M, ŠTAMPAR F, Hudina M. Contents of sugars and organic acids in the cultivars of peach (*Prunus persica* L.) and nectarine (*Prunus persica* var. nucipersica Schneid). Acta agriculturae Slov. 2004;83(1):53–61.

[18] Abidi W, Moreno Sánchez MÁ, Gogorcena Aoiz Y. Phenotypic and biochemical diversity in peach (*Prunus persica* L.) Batsch] cultivars. J new Sci Agric Biotechnol. 2018;51(5):3171–8.

[19] Jayarajan S, Sharma RR, Sethi S, Saha S, Sharma VK, Singh S. Chemical and nutritional evaluation of major genotypes of nectarine (*Prunus persica* var nectarina) grown in North-Western Himalayas. J Food Sci Technol. 2019;56(9):4266–73. 10.1007/s13197-019-03896-2 PMC6706499 31477997

[20] Küçük O, Muradoğlu F. Pomological and chemical properties of some peach (*Prunus persica* L.) cultivars grown in Mustafakemalpaşa (BURSA). Bahçe. 2020;49(1):241–5.

[21] Mosie T, Setu H, Seleshi G. Physicochemical quality properties of peach (*Prunus persica* L.) varieties at Holeta, Ethiopia. Fruit Grow Res. 2024. 10.33045/fgr.v40.2024.28.

[22] Yaman M, Remzi UĞUR, Sümbül A, Yusuf KEÇE, Gönültaş M, Ünsal HT, Yilmaz KU. Determination of fruit characteristics, nutrients and biochemical contents of Transvalia (*Prunus persica* L.) peach cultivar grafted on different clonal rootstocks obtained by selection and hybridization. Sci Hort. 2024;330:113093.

[23] Taheri G, Hajnajari H. Evaluation of 22 peach cultivars in Moghan (Iran) climatic conditions. In *Mеждународная дистанционная научная конференция «Интенсификация плодоводства Беларуси: Традиции, достижения, перспективы» Минский р-н, Беларусь*. 2010.

[24] Milošević T, Milošević N, Glišić I. Evaluation of fruit growth and postharvest physical and chemical properties of nectarine [*Prunus persica var*. nectarina (Ait.) Maxim]. Acta Scientiarum Polonorum Hortorum Cultus. 2012;11(5):17–30.

[25] Ghrab M, Zitouna R, Masmoudi MM, Ben Mechlia N. Phenology and yield efficiency of early, mid-, and late-maturing cultivars of peach in irrigated orchards under Mediterranean climate. Int J Fruit Sci. 2016;16(3):323–34.

[26] Rencuzoğulları E, Dikbaş O, Çalışkan O. Fruit Quality Characteristics of Flariba Nectarine (*Prunus persica* var. nectarina Maxim) Cultivar Grown Under Protected Cultivation. Bahçe. 2016;45(1):1054–8.

[27] Srivastava KK, Rajan S, Kumar D, Singh SR, Verma HC. Growth pattern and phenological stages of peach (*Prunus persica*) under subtropics. Indian J Agric Sci. 2020;90(2):356–60.

[28] Çalışkan O, Bayazit S, Gündüz K, Kilic D, Göktaş S. Earliness, yield, and fruit quality characteristics in low chill peach-nectarines: a comparison of protected and open area cultivation. Turkish J Agric Forestry. 2021;45(2):191–202.

[29] Demiral S, Ülger S. Investigation of the effects of different applications on earliness and fruit properties of some peach cultivars grown in pots in plastic greenhouse. Mediterranean Agricultural Sci. 2021;34(1):1–7.

[30] Ganji Moghadam E, Ghahremani A, Seyed Masoumi Khayavi SY. Evaluation of pomological and morphological traits of some Peach (*Prunus persica* L. BatSch) cultivars and genotypes under Khorasan Razavi climatic conditions. J Hortic Sci. 2021;35(1):87–102.

[31] Koşar DA, Koşar MB, Ertürk U. Investigation of phenological and pomological characteristics of some flatt peach and nectarine cultivars in Bursa (Türkiye) Conditions. KSU J Agric Nat. 2023;26(4):722–31.

[32] Çalışkan O, Kilic D, Bayazit S. Fruit yield and quality performance of low chilling nectarine cultivars under Mediterranean climate. J Agricultural Sci Technol. 2024;26(3):637–53.

[33] Gur E, Gundogdu MA, Yilmaz N, Seker M. Assessing the Aromatic and Quality Components of a Novel Peach Genotype (‘Sırrı’) Grafted onto Various Rootstocks in the Lapseki Area. Horticulturae. 2025;11(4):364.

[34] Gülsoy E, Kus E, Tan B, Alwazeer D, Tan K. Physico-chemical Changes and Optimal Harvest Determination of ‘Zeferan’Peach (*Prunus Persica* L.) Cultivar Throughout the Fruit Ripening Period. Appl Fruit Sci. 2025;67(3):163.

[35] Kaya Ç, Şeker M. Investigation of Pomological and Morphological Traits of Some Peach (*Prunus persica* L.) Cultivars and Local Genotypes Grown in Çanakkale Ecological Conditions. J Science-Technology-Innovatio Ecosyste. 2025;6(1):41–52.

[36] Demiral S. Cultivation of Some Stone Fruit Species in Pots for the Purpose of Achieving Earliness. PhD Thesis, Graduate School of Natural and Applied Sciences, Department of Horticulture, Akdeniz University, Türkiye. 2015;226 p.

[37] Liu S, Xu Q, Li X, Wang Y, Zhu J, Ning C, Meng X. Effects of high hydrostatic pressure on physicochemical properties, enzymes activity, and antioxidant capacities of anthocyanins extracts of wild Lonicera caerulea berry. Innovative food Sci Emerg Technol. 2016;36:48–58.

[38] Dincer C, Topuz A, Sahin-Nadeem H, Ozdemir KS, Cam IB, Tontul I, Ay ST. A comparative study on phenolic composition, antioxidant activity and essential oil content of wild and cultivated sage (*Salvia fruticosa* Miller) as influenced by storage. Ind Crops Prod. 2012;39:170–6.

[39] Asami DK, Hong YJ, Barrett DM, Mitchell AE. Comparison of the total phenolic and ascorbic acid content of freeze-dried and air-dried marionberry, strawberry, and corn grown using conventional, organic, and sustainable agricultural practices. J Agric Food Chem. 2003;51(5):1237–41. 10.1021/jf020635c 12590461

[40] Tetik N, Turhan I, Oziyci HR, Karhan M. Determination of d-pinitol in carob syrup. Int J Food Sci Nutr. 2011;62(6):572–6. 10.3109/09637486.2011.560564 21466274

[41] Lee H, Huang CC, Lin SY. Phenology-Informed Strategies for Climate-Resilient Peach Production: Shoot Growth, Leaf Fall, and Flowering of Two Low-Chill Cultivars in Humid Subtropical Central Taiwan. Agronomy. 2025;15(12):2748.

[42] Erez A. Bud dormancy; phenomenon, problems and solutions in the tropics and subtropics. In Temperate fruit crops in warm climates. Dordrecht: Springer Netherlands. 2000;17–48. 10.1007/978-94-017-3215-4_2.

[43] Yong LI, Ke CAO, Chang-wen CHEN, Xin-wei WANG, Li-rong WANG. Accumulated chilling hours during endodormancy impact blooming and fruit shape development in peach (*Prunus persica* L). J Integr Agric. 2016;15(6):1267–74.

[44] Sarkhosh A, Olmstead M, Williamson J, Chaparro J, Popenoe J. Alternative opportunities for small farms: Peach and nectarine production review. Univ Fla IFAS Ext. 2023; https://edis.ifas.ufl.edu/publication/AC018.

[45] Reig G, Iglesias I, Echeverría G. Agronomical performance, fruit quality and sensory attributes of several flat peach and flat nectarine cultivars. In VII International Peach Symposium. 2009;962,563–9. 10.17660/ActaHortic.2012.962.76.

[46] Lopez G, DeJong TM. Spring temperatures have a major effect on early stages of peach fruit growth. J Hortic Sci Biotechnol. 2007;82(4):507–12.

[47] Farina V, Lo Bianco R, Mazzaglia A. Evaluation of late-maturing peach and nectarine fruit quality by chemical, physical, and sensory determinations. Agriculture. 2019;9(9):189.

[48] Sejfić JH, Aliman J, Hadžiabulić S, Jukić AŠ, Badžak N, Skender A, Dorbić B. Morphological and chemical analysis introduced of the nectarine cultivars in Herzegovina. Pomologia Croatica. 2023;27. 10.33128/pc.27.1-4.1.

[49] Gündoğdu MA, Gür E, Şeker M. Determination of Some Pomological Characteristics of Different Nectarine Varieties Cultivated in Lapseki Ecology. Bahçe. 2024;53(1):327–32.

[50] Lee J, Jung K, Kim D. Comparative analysis of phenolic compounds in peach and nectarine cultivars. Sci Hort. 2021;288:110365.

[51] Özkan M, Demir S, Yıldız F. Phenolic content and antioxidant activity of different peach varieties. Turkish J Agric Forestry. 2019;43(2):105–14.

[52] Çelik M, Yılmaz H, Kaya C. Variation in flavonoid and phenolic content among peach and nectarine cultivars. J Hortic Sci. 2020;95(3):245–54.

[53] Hajilou J, Fakhimrezaei S. Evaluation of fruit physicochemical properties in some peach cultivars. Res Plant Biology. 2011;1(5):16–21.

[54] Wang L, Zhang Y, Xu J. Vitamin C and antioxidant profiles of peach and nectarine cultivars: Variation and implications for human health. Food Sci Technol. 2020;40(2):347–56.

[55] Gür E, Gündoğdu MA, Şeker M. Determination of Pomological Characteristics of Peach Varieties Extensively Cultivated in Lapseki Ecology. ÇOMÜ LJAR. 2020;1(2):90–100.

[56] Brooks SJ, Moore JN, Murphy JB. Quantitative and qualitative changes in sugar content of peach genotypes [*Prunus persica* (L.) Batsch]. J Am Soc Hortic Sci. 1993;118(1):97–100.

[57] Gao F, Wang S, Li Q, Zhang H. Vitamin C content and antioxidant capacity in different peach (*Prunus persica* L.) cultivars. Food Chem. 2017;221:1282–9.

